# Protein Expression Analysis of an In Vitro Murine Model of Prostate Cancer Progression: Towards Identification of High-Potential Therapeutic Targets

**DOI:** 10.3390/jpm10030083

**Published:** 2020-08-10

**Authors:** Hisham F. Bahmad, Wenjing Peng, Rui Zhu, Farah Ballout, Alissar Monzer, Mohamad K. Elajami, Firas Kobeissy, Wassim Abou-Kheir, Yehia Mechref

**Affiliations:** 1 Department of Anatomy, Cell Biology and Physiological Sciences, Faculty of Medicine, American University of Beirut, Beirut 1107-2020, Lebanon; hfbahmad@gmail.com (H.F.B.); frb03@mail.aub.edu (F.B.); aam48@mail.aub.edu (A.M.); Mohamadelajami@hotmail.com (M.K.E.); 2 Arkadi M. Rywlin M.D. Department of Pathology and Laboratory Medicine, Mount Sinai Medical Center, Miami Beach, FL 33140, USA; 3 Herbert Wertheim College of Medicine, Florida International University, Miami, FL 33199, USA; 4 Department of Chemistry and Biochemistry, Texas Tech University, Lubbock, TX 79409, USA; Wenjing.Peng@ttu.edu (W.P.); Rui.Zhu@ttu.edu (R.Z.); 5 Department of Internal Medicine, Mount Sinai Medical Center, Miami Beach, FL 33140, USA; 6 Department of Biochemistry and Molecular Genetics, Faculty of Medicine, American University of Beirut, Beirut 1107-2020, Lebanon

**Keywords:** prostate cancer, differential expression analysis, progression, proteomics, LC-MS/MS, signaling pathways, therapeutic target

## Abstract

Background: Prostate cancer (PC) is the most frequently diagnosed cancer among men worldwide. The poor prognosis of PC is largely due to late diagnosis of the disease when it has progressed to advanced stages marked by androgen-independence. We interrogated proteomic signatures that embody the transition of PC from an androgen-dependent (AD) to an androgen-independent (AI) state. Methods: We have previously established AD and AI murine PC cell lines, PLum-AD and PLum-AI, respectively, which recapitulate primary and progressive PC at phenotypic and subcellular levels. We statistically surveyed global protein expression profiles in these cell lines. Differential profiles were functionally interrogated by pathways and protein–protein interaction network analyses. Results: Protein expression pattern analysis revealed a total of 683 proteins, among which 99 were significantly differentially altered in PLum-AI cells as compared to PLum-AD cells (45 increased and 54 decreased). Principal component analysis (PCA) revealed that the two different cell lines clearly separated apart, indicating a significant proteome expression difference between them. Four of the proteins (vimentin, catalase, EpCAM, and caspase 3) that were differentially expressed in PLum-AI cells compared to PLum-AD cells were subjected to biochemical validation by Western blotting. Biological process gene ontology (GO) analysis of the differentially expressed proteins demonstrated enrichment of biological functions and pathways in PLum-AI cells that are central to PI3 kinase and androgen receptor pathways. Besides, other relevant biological processes that are enriched in PLum-AI cells included cell adhesion and cell migration processes, cell and DNA damage, apoptosis, and cell cycle regulation. Conclusions: Our protein expression analysis of a murine in vitro model of PC progression identified differential protein spots that denote this progression and that comprise high-potential targets for early treatment of PC with a personalized patient-specific approach. Efforts are underway to functionally assess the potential roles of these proteins as therapeutic targets for PC progression.

## 1. Introduction

Prostate cancer (PC) is the most frequently diagnosed cancer among men worldwide and the second leading cause of male deaths from cancer globally [1]. It is an indolent tumor that grows unhurriedly but poses significant threat to patients’ lives on the long-term [2]. Evidence showed that during early androgen-dependent stages of the disease, tumor cells are mainly influenced by androgen production as a major mediator for their growth and survival using several axes [3,4,5,6]; therefore, patients with primary androgen-dependent PC respond well to androgen-deprivation therapy (ADT) [5,7,8]. However, the disease progresses over time in around one fifth of patients to a metastatic and advanced androgen-independent stage known as castration-resistant prostate cancer (CRPC) [9]. Upon progression, tumor cells tailor different cellular pathways and mechanisms to survive the androgen-depleted environment [8]. Proven mechanisms of such adaptation include androgen receptor (AR) gene amplification, AR gene mutations, involvement of AR co-regulators such as AR-associated proteins, ligand-independent activation of the AR, and the involvement of cancer stem cells (CSCs) [10,11,12,13].

Management of advanced PC propounds substantial challenges and various therapeutic approaches are then considered including radical prostatectomy (RP) surgery, chemotherapy, and radiation therapy [14]. In this regard, the success rates of the different therapeutic modalities used for treating PC can be greatly improved if the disease is diagnosed at an early stage [15]. Currently, there are no reliable and effective biomarkers for PC that can specifically distinguish patients from healthy individuals, and patients who need aggressive therapy to stop progression of their disease from those who should avoid overtreatment [15,16], which paves the way to personalized patient-specific therapy. Therefore, efforts have been made in order to identify the parameters that accurately predict the prognosis and clinical outcome following RP, which can greatly aid in planning for the appropriate postoperative therapy that should be used in each patient [17]. Those parameters include clinicopathological factors, such as prostate specific antigen (PSA), Gleason score (GS), and pathological stage, among others [18,19,20,21].

PC progression from androgen-dependence (AD) to androgen-independence (AI) is tightly linked to dismal prognosis, warranting the need for new strategies for early detection and treatment of progressive PC. Detecting the molecular signatures and proteomic aberrations pertaining to this progression will greatly help in understanding the disease and shaping its management accordingly [22,23]. In this regard, protein expression analysis enables the identification of pathways and biological processes that are aberrantly modulated in human diseases and specific phenotypes [24,25,26,27,28,29,30], thus providing a viable framework for underscoring potential biomarkers and therapeutic drug targets [26,31]. Proteomic analysis also offers the capability to screen and uncover the expression levels of tens and hundreds of proteins simultaneously and objectively [32].

We have recently established two murine PC cell lines that represent AD (PLum-AD) and AI (PLum-AI) PC [12]. However, we have a poor understanding of the mechanisms underlying the clinical progression of PC. Therefore, using proteomic analysis and a novel model which we have previously developed signifying the development of AI, we aimed to fill these voids, first by understanding global signaling cues in the progression of AD-to-AI PC and next by identifying from these cues candidate biomarkers for early human PC progression. Such proteomic-based approaches will help researchers set novel strategies to better understand PC and treat PC patients, inaugurating a new era of “personalized medicine”.

## 2. Materials and Methods

### 2.1. Cell Lines, Culture Conditions, and Reagents

Mouse PLum-AD (androgen-dependent) and PLum-AI (androgen-independent) PC cells, that were established in our laboratory, were cultivated in PrEBM™ prostate epithelial cell growth basal medium (Lonza, Switzerland; cat #CC-3165) supplemented with PrEBM™ SingleQuots™ supplements (Lonza, Switzerland; cat #CC-4177), as previously described [12]. PLum-AD cells grew in serum-free media while 5% of heat-inactivated fetal bovine serum (FBS; Sigma-Aldrich, St. Louis, MO, USA; cat #F9665) was added to the culture medium of PLum-AI cells. For both cell lines, media was supplemented with 1% penicillin/streptomycin (Biowest, Nuaillé, France; cat #L0022-100) and Plasmocin™ prophylactic (Invivogen; cat #ant-mpp). Cells were incubated at 37 °C in a humidified incubator containing 5% CO_2_.

Cells were seeded in triplicates in 75 cm^2^ plates at a density of 5 × 10^5^ cells per plate and cultured for 6–10 days until reaching 80% confluency. Cells were then washed twice with 10 mL of Dulbecco’s phosphate buffered saline (D-PBS) (Sigma-Aldrich, St. Louis, MO, USA; cat #D8537-500ML) and detached by 2.5 mL trypsin–ethylenediaminetetraacetic acid (EDTA) solution (Sigma-Aldrich, St. Louis, MO, USA; cat #T4049). Then, 2.5 mL of fresh culture medium was added to neutralize trypsin. Harvested cells were centrifuged at 900 rpm for 5 min and cell pellets were collected, washed twice with D-PBS, and stored at −20 °C for proteomic analysis.

Formic acid (FA; Sigma-Aldrich, St. Louis, MO, USA; cat #F0507), 1,4-dithiothreitol (DTT; Sigma-Aldrich, St. Louis, MO, USA; cat #D0632), iodoacetamide (IAA; Sigma-Aldrich, St. Louis, MO, USA; cat #I1149), ammonium bicarbonate (ABC; Sigma-Aldrich, St. Louis, MO, USA; cat #09830), and sodium deoxycholate (SDC; Sigma-Aldrich, St. Louis, MO, USA; cat #D6750) were obtained from Sigma-Aldrich (St. Louis, MO, USA). Trypsin/Lys-C mix, mass spectrometry grade was obtained from Promega (Madison, WI, USA; cat #V5071). High-performance liquid chromatography (HPLC) grade methanol (Cat #A452-1), acetonitrile (Cat #A21-1), and water (Cat #W71) were obtained from Thermo Fisher Scientific (San Jose, CA, USA).

The study with all its experimental protocols was conducted under the Institutional Review Board (IRB) approval of the American University of Beirut (AUB) (Date: March 2020; project identification code: WAK2020). The work described herein has been carried out in accordance with relevant guidelines and regulations.

### 2.2. Protein Extraction, Denaturation, and Digestion

Three biological replicates of cells from PLum-AD and PLum-AI cell lines were suspended in 200 μL of 50 mM ABC buffer (pH 8.0) with 5% SDC and lysed using a beads beating homogenizer (Benchmark Scientific, Edison, NJ, USA) at 4 °C [33]. The beads beating homogenizer was set to 4 rounds, shaking 30 s for each round with a 30 s break between each round to reduce the heat. After beads beating, samples were sonicated using an ultrasonic bath (Thermo Fisher Scientific, San Jose, CA, USA) for 1 h. While sonicating, ice was added into the water to keep the cold temperature (adding ice periodically to ensure they were not completely melted). After sonication, samples were centrifuge at 1000× *g* for 10 min. Supernatants were collected. Then, a 2 μL of cell lysate was taken out to determine the protein concentration through a Micro BCA Protein Assay Kit (Thermo Fisher Scientific, San Jose, CA, USA). The remaining samples were denatured at 90 °C for 15 min and reduced by 5 mM DTT at 60 °C for 45 min. After reduction, samples were alkylated by IAA at 37 °C for 45 min in the dark. Then, another 5 mM DTT was added to the samples and incubate at 37 °C for 30 min to quench the alkylation reaction. Next, additional ABC buffer was added to the sample to adjust the final concentration of SDC to 0.5%. Then, trypsin/Lys-C mix was added following a 1/25 (enzyme/protein, µg/µg) ratio, and incubated at 37 °C in a water bath for 18 h. After tryptic digestion, 1% FA (final concentration) was added to the samples and vortex thoroughly to precipitate SDC. Then, samples were centrifuged at 21,100× *g* for 10 min to remove SDC. Supernatants containing digested peptides were dried and ready to be analyzed by LC-MS/MS.

### 2.3. Liquid Chromatography (LC)–Mass Spectrometer (MS)/MS Analysis

Peptides samples were resuspended in 2% acetonitrile (ACN) (with 0.1% FA) solution and centrifuged at 21,100× *g* for 10 min before injecting to LC-MS/MS. A Dionex Ulitimate 3000 nanoLC system (Thermo Fisher Scientific, San Jose, CA, USA) and a Linear Trap Quadropole (LTQ) Orbitrap Velos mass spectrometer (Thermo Fisher Scientific, San Jose, CA, USA) were utilized for the proteomic analysis. LC was interfaced with MS via a nanoESI source. Peptides digested from 1 μg of proteome were injected for each sample. An online purification was performed using a trap column (Acclaim PepMap 100 C18, 75 µm I.D. × 2 cm, 3 µm particle sizes, 100 Å pore sizes, Thermo Scientific, San Jose, CA, USA) to remove possible salts and trap the peptides. The separation of peptides was performed on an Acclaim PepMap C18 column (75 µm I.D. × 15 cm, 2 µm particle sizes, 100 Å pore sizes, Thermo Fisher Scientific, San Jose, CA, USA). A 120 min gradients was utilized to separate peptides. The column temperature was set to 29.5 °C. Mobile phase A was 2% ACN in water with 0.1% FA, while mobile phase B was 100% ACN with 0.1% FA. The gradient of mobile phase B was set as following: 0–10 min, 5% B; 10–65 min, 5–20% B; 65–90 min, 20–30% B, 90–110 min, 30–50% B; 110–111 min, 50–80% B; 111–115 min, 80% B; 115–116 min, 80–5% B, and 116–120 min, 5% B.

The resolution of full MS was set to 60,000 with the *m*/*z* range of 400–2000. Collision-induced dissociation (CID) was performed for MS/MS scan with a normalized collision energy of 35%, Q-value of 0.25, and activation time of 10 ms. A data-dependent acquisition mode was utilized. The top 10 most intense ions observed in the full MS scan were selected to conduct MS/MS scan. A repeat count of 2, repeat duration of 30 s, exclusion list size of 200, and exclusion duration of 90 s was set for dynamic exclusion.

### 2.4. Protein Identification and Quantification

LC-MS/MS data were first converted to a general format (*.mgf) using Proteome Discover software, and search against a UniProt database (2014_06, Mus musculus, 16,677 entries) using Mascot software (Matrix Science Inc., Boston, MA, USA). Carbamidomethylation of cysteine was set to be the fix modification while oxidation of methionine was the variable modification. The *m*/*z* tolerance of full MS was set to 6 ppm. The *m*/*z* tolerance of MS/MS was 0.8 Da. Maximum peptide miss cleavage was set to 2. Peptides identified by Mascot were further verified and quantified using Scaffold software. The peptide and protein identification probabilities were set to 95% and 99%, respectively. A protein identification was accepted only when it contained more than 2 identified peptides. Spectra count was employed as a protein quantitation method. Normalized quantitative values were used to represent the expression levels of proteins in each sample. After Scaffold quantitation, a secondary filter was added to keep the proteins that were detected in at least two replicates.

### 2.5. Bioinformatics Analysis of the Differentially Abundant Proteins

We used the Search Tool for the Retrieval of Interacting Genes/Proteins database (STRING v11.0) [34] to construct the protein–protein interaction (PPI) network associated with the differentially expressed proteins in PLum-AI vs. PLum-AD cells, by inputting the protein spots into the STRING database (https://string-db.org/). We then determined the relationships among the differentially expressed proteins in PLum-AI cells vs. PLum-AD cells via The Elsevier’s Pathway Studio version 10.0 (Ariadne Genomics, Elsevier) and the Ariadne ResNet database [35,36]. “Subnetwork Enrichment Analysis” (SNEA) was pursued to identify the biological and functional pathways that display statistically significant alterations in PLum-AI cells vs. PLum-AD cells. 

### 2.6. Western Blot Analysis

Cellular protein extracts were prepared in Radio-Immunoprecipitation Assay (RIPA) lysis buffer (Santa Cruz, CA, USA; cat #sc-24948). Protein extracts were quantified using the DC Bio-Rad Protein Assay (Bio-Rad Laboratories, Hercules, CA, USA) according to the manufacturer’s protocol. Protein samples were mixed with 10% β-mercaptoethanol and 2X Laemmli sample buffer (Bio-Rad, CA, USA) for gel electrophoresis. An equal amount of protein lysate was separated on 10% sodium dodecyl sulfate–polyacrylamide gel electrophoresis (SDS–PAGE) for 2h at 90 V then transferred onto 0.45μm nitrocellulose membrane (Bio-Rad, CA, USA) in transfer buffer overnight at 40 °C. Membranes were blocked with 5% skim milk in tris-buffered saline with 0.1% tween 20 (TBST) for 1 h and then incubated overnight at 4 °C with rabbit polyclonal anti-vimentin (1:50 dilution; Santa Cruz Biotechnology, CA, USA), rabbit monoclonal anti-EpCAM (1:200 dilution; Abcam Inc., Cambridge, MA, USA; cat #ab32392), mouse monoclonal anti-catalase (1:1000 dilution; Sigma-Aldrich, St. Louis, MO, USA; cat #C0979), and rabbit monoclonal anti-caspase 3 (1:500 dilution, Cell Signaling Technology, Danvers, MA, USA; cat #9662S). Membranes were then washed three times with TBST and incubated with the diluted (1:1000) Horseradish Peroxidase (HRP)-conjugated secondary antibody (goat anti-mouse (cat #sc-516102) and mouse anti-rabbit (Santa Cruz Biotechnology, CA, USA; cat #sc-2357)) for 1 h at room temperature. Hybridization with glyceraldehyde 3-phosphate dehydrogenase (GAPDH)-HRP (6C5) (1:10,000–20,000; Abnova, Taipei, Taiwan; cat #MAB5476) coupled antibody was performed for 30 min at room temperature as housekeeping gene. Target proteins were detected using the Enhanced Chemiluminescence (ECL) system (Bio-Rad, CA, USA). Images were generated and quantified using ChemiDoc™ Imaging Systems (Bio-Rad, CA, USA).

### 2.7. Statistical Analysis

One-way ANOVA was employed to investigate the statistical differences between PLum-AD and PLum-AI cell groups (*n* = 3) using IBM Statistical Package for the Social Sciences (SPSS) Statistics ver. 20.0 (IBM Co., Armonk, NY, USA). Protein expressions were considered altered when *p*-values were less than 0.05. Fisher’s test was used for SNEA to look for nonrandom associations between the two categorical variables. Data significance in the Western blot experiments was determined using Student’s *t*-test. *p*-values of *p* < 0.05 (*) and *p* < 0.01 (**) were labeled significant and highly significant, respectively.

## 3. Results

### 3.1. Protein Expression Profiles of PLum-AI vs. PLum-AD Cell Lines

**Figure 1:**
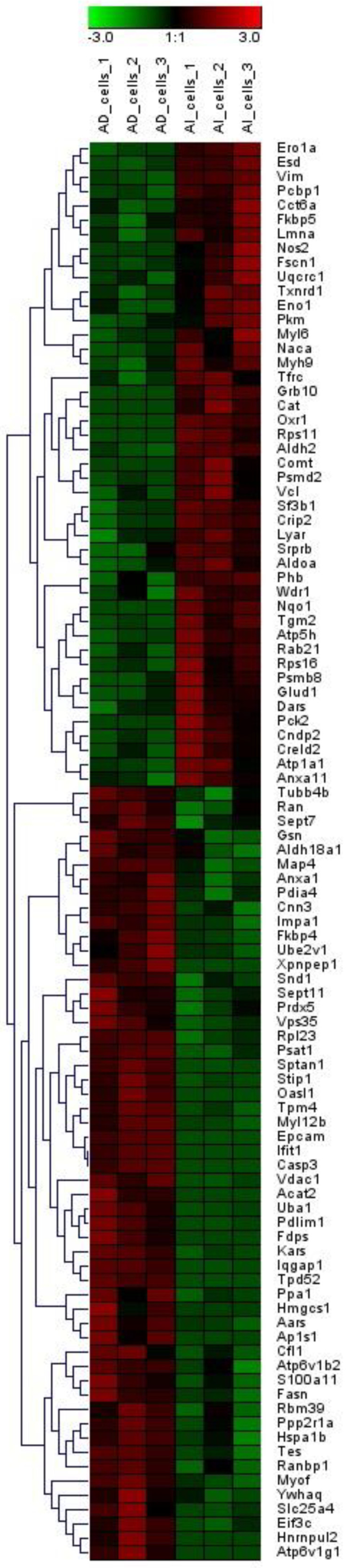
Heatmap of proteins whose expressions exhibited significant differences between PLum-AI and PLum-AD cells. Expression alterations were observed among these proteins.

LC-MS/MS-based bottom-up proteomics was performed for each cell line. A total of 683 proteins were identified. Proteins that identified in only one replicate were filtered out, and the remaining proteins were utilized for the differential expression analysis. One-way ANOVA was employed to estimate the statistical significance of the differences between PLum-AI and PLum-AD cell lines. Any proteins with *p* < 0.05 were considered to have the expression change. Overall, 99 proteins exhibited the expression changes between PLum-AI and Plum-AD cell lines (Table 1, Supporting Information Table S1). Figure 1 depicts the expression discrepancy of these proteins.

Compared to PLum-AD cells, 45 proteins were upregulated in PLum-AI cells while 54 proteins were downregulated. Among them, 8 proteins (Nos2, Oxr1, Pck2, Grb10, Cat, Nqo1, Comt, and Rps11) were only identified in PLum-AI cells (and not in PLum-AD cells) while 12 proteins (Hnrnpul2, Xpnpep1, Hmgcs1, Oasl1, Ifit1, Acat2, Fdps, Epcam, Casp3, Tpd52, Ap1s1, and Atp6v1g1) were exclusively identified in PLum-AD cells (and not in PLum-AI cells).

### 3.2. Unsupervised Principal Component Analysis (PCA)

**Figure 2:**
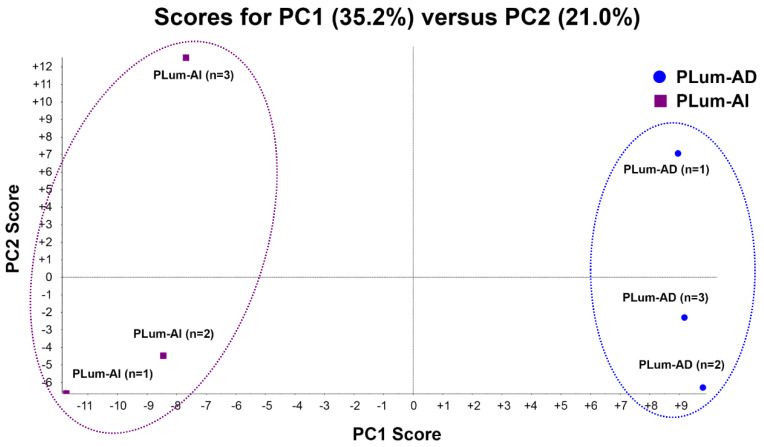
Principle component analysis (PCA) of protein profiles. PCA projection of protein profiles of PLum-AI and PLum-AD cells obtained from three independent biological samples for each cell line.

PCA is a mathematical method that decreases the dimensions of a complex dataset which contains a series of related independent variables. It can convert the data to a set of principal components through an orthogonal transformation to display the similarity of data groups by plotting points on a map [37]. Figure 2 depicts the PCA analysis of the two cell lines, including their triplicates. Plots having the same color and shape represent the same cell line. The difference between each sample can be observed via primary principal component (PC1) and secondary principal component (PC2). Three triplicates of the same cell line are clustered together, suggesting a satisfactory reproducibility of LC-MS/MS based proteomic analysis in this study. Two different cell lines are clearly separated apart, indicating a significant proteome expression difference between these two cell lines.

### 3.3. Validation of Some of the Differentially Expressed Proteins in PLum-AI vs. PLum-AD Cells

**Figure 3:**
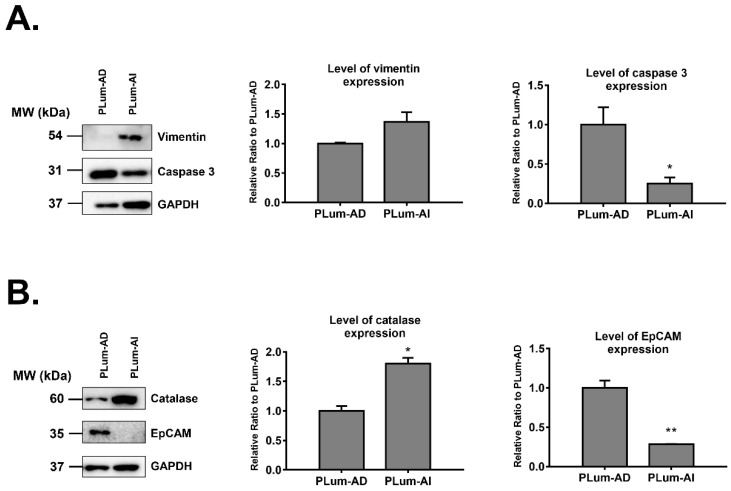
Western blot validation of four proteins identified by mass spectrometry to be differentially expressed in PLum-AI relative to PLum-AD cells (*n* = 3). (**A**) Western blot validation of vimentin and caspase 3 proteins. (**B**) Western blot validation of catalase and EpCAM proteins. Bands were detected by enhanced chemiluminescence (ECL) using the ChemiDoc MP Imaging System. Protein expression was quantified using Image Lab software, relative to the expression of glyceraldehyde 3-phosphate dehydrogenase (GAPDH), a housekeeping gene equally expressed in treated and non-treated cells/spheres. Results are expressed as relative ratio to control. Data represent an average of three independent experiments. The data are reported as mean ± SEM. (* p < 0.05, ** p < 0.01; PLum-AI cells compared to PLum-AD cells, student independent t-test).

Four of the proteins that were differentially expressed in PLum-AI cells compared to PLum-AD cells were subjected to biochemical validation by Western blotting. We selected two proteins that were found to be highly upregulated (catalase and vimentin) and two proteins that were highly downregulated (EpCAM and caspase 3) in PLum-AI cells. Selection of those four proteins depended on the availability of their antibodies, literature relevance, and their levels of differential expression. Western blotting results were consistent with the proteomics data where analyses revealed a statistically significant increase in protein expression of catalase and vimentin (*p* < 0.05; student’s *t*-test) and a decrease in expression of caspase 3 and EpCAM (*p* < 0.05; student’s *t*-test) in PLum-AI cell samples when compared to PLum-AD cells (Figure 3). The densitometry readings/intensity ratio of each band in addition to whole blot (uncropped blots) are included in Supporting Information Figure S1.

### 3.4. Construction of the Protein–Protein Interaction (PPI) Network Associated with the Differentially Expressed Proteins in PLum-AI vs. PLum-AD Cells

**Figure 4:**
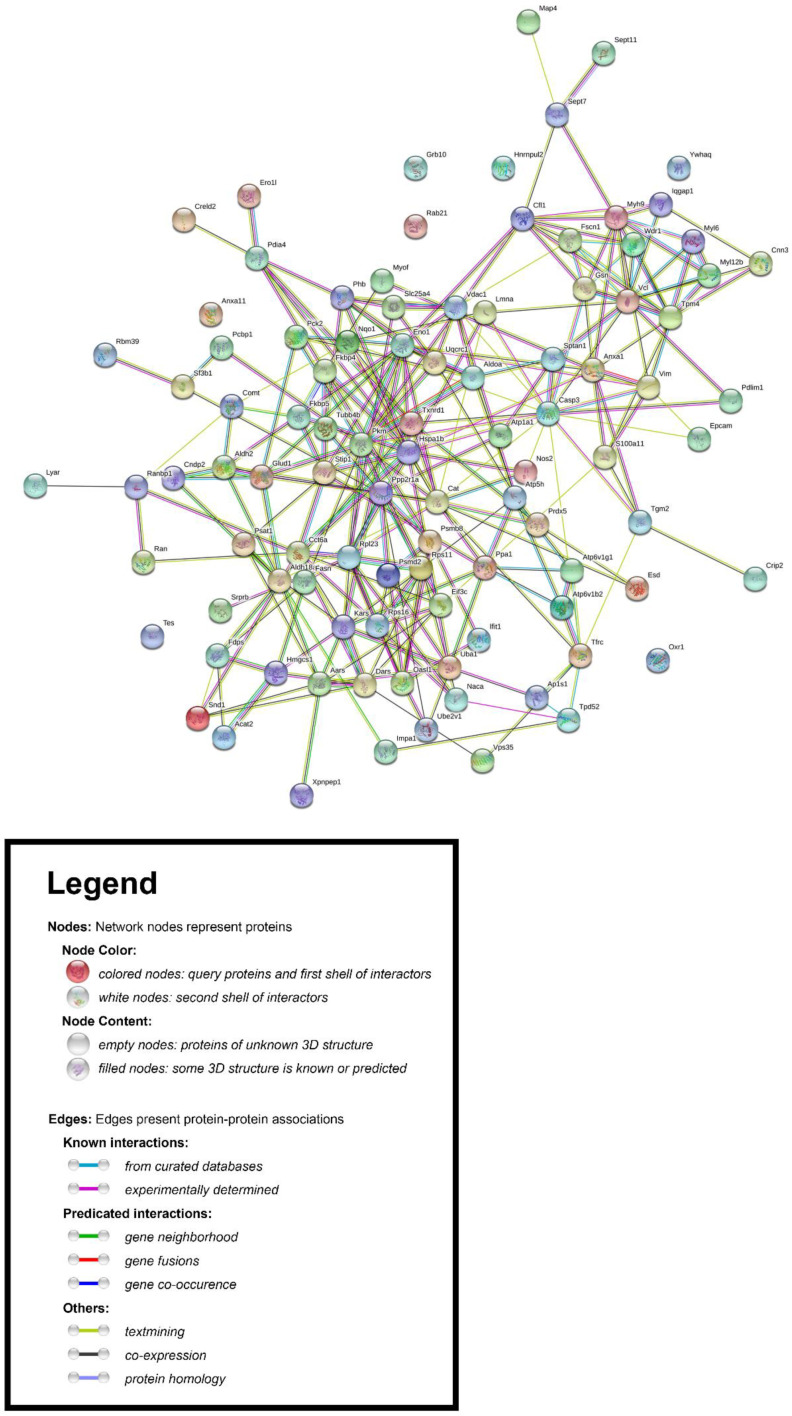
Protein–protein interaction (PPI) network associated with the differentially expressed proteins in PLum-AI vs. PLum-AD cells. The Retrieval of Interacting Genes/Proteins database (STRING v11.0) [34] was used to construct the PPI network.

Using the Search Tool for the Retrieval of Interacting Genes/Proteins database (STRING v11.0) [34], we constructed the protein–protein interaction (PPI) network associated with the differentially expressed proteins in PLum-AI vs. PLum-AD cells. We inserted the list of 99 differentially expressed protein spots as input and allowed the STRING database (https://string-db.org/) to search for neighbor interactors and proteins that possess interactions with the inputted proteins. The PPI network was then built involving all proteins and interactions between them (Figure 4).

### 3.5. Subnetwork Analyses of Pathways Associated with the Differentially Expressed Proteins in PLum-AI vs. PLum-AD Cells

**Figure 5:**
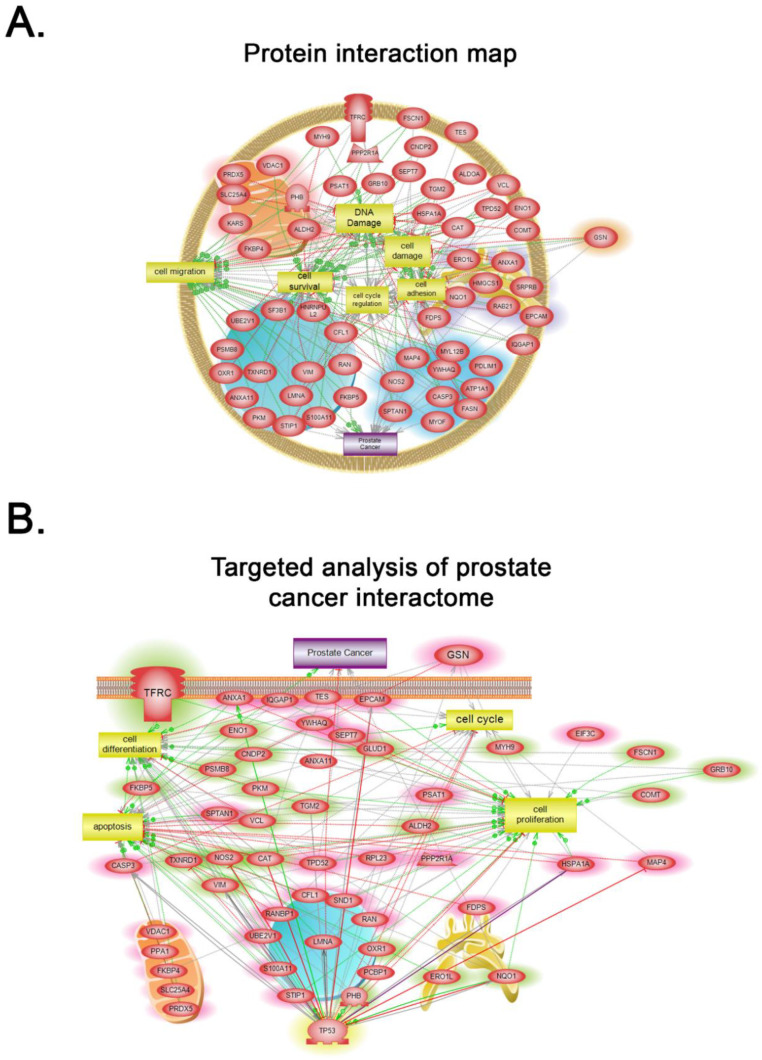
Systems biology analysis. (**A**) Biologically statistically significant protein interaction map showing enrichment of biological functions and pathways in PLum-AI cells that are central to cell migration, cell cycle regulation, cell damage, cell survival, DNA damage, and cell adhesion (Supporting Information Table S2). (**B**) Targeted analysis of prostate cancer interactome in PLum-AI cells vs. PLum-AD cells revealed dysregulation of proteins involved in cell differentiation, cell proliferation, cell cycle, and apoptosis (Supporting Information Table S3). Relationships among the differentially expressed proteins in PLum-AI cells vs. PLum-AD cells were determined using Elsevier’s Pathway Studio version 10.0 (Ariadne Genomics, Elsevier) and the Ariadne ResNet database [35,36].

**Figure 6:**
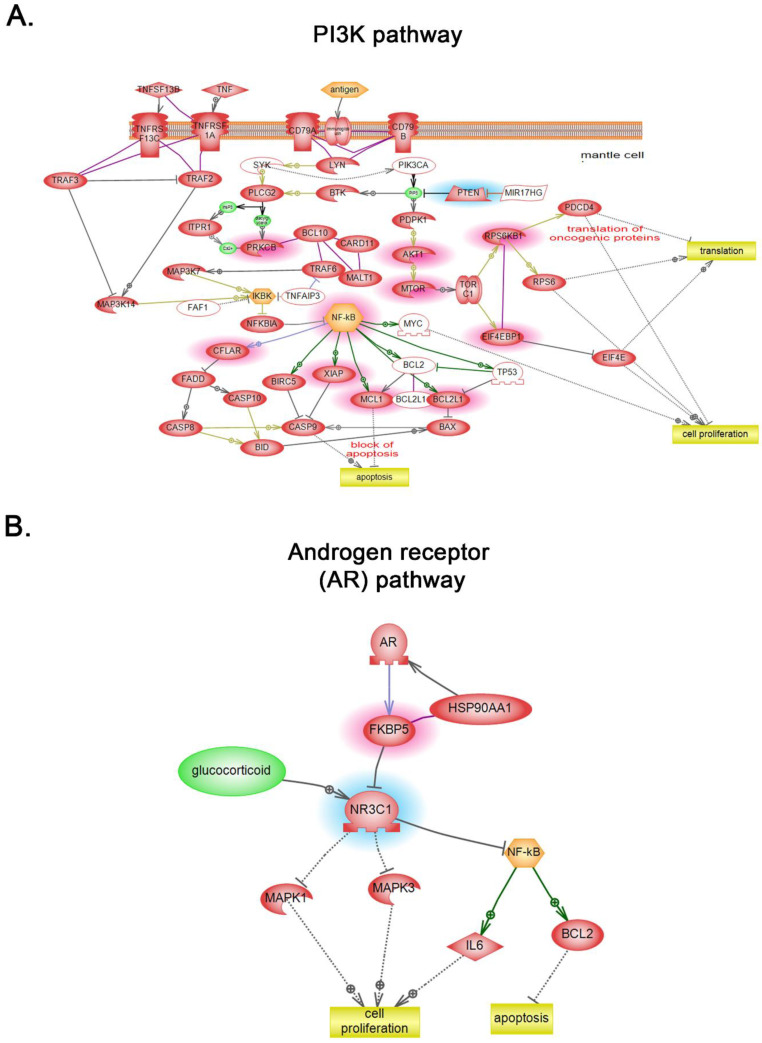
Global subnetwork analyses. Biological process gene ontology (GO) enrichment analysis of the differentially expressed proteins demonstrated enrichment of biological functions in PLum-AI cells that are central to PI3 kinase (**A**) and androgen receptor (**B**) pathways (Supporting Information Tables S4 and S5, respectively). Relationships among the differentially expressed proteins in PLum-AI cells vs. PLum-AD cells were determined using Elsevier’s Pathway Studio version 10.0 (Ariadne Genomics, Elsevier) and the Ariadne ResNet database [35,36].

Global subnetwork analyses of PLum-AI cells in comparison to PLum-AD cells showed differences in the involvement of protein pathways relevant to PC progression (Figure 5 and Figure 6). Indeed, biologically statistically significant protein interaction analysis showed enrichment of biological functions and pathways in PLum-AI cells that are central to cell migration, cell cycle regulation, cell damage, cell survival, DNA damage, and cell adhesion (Figure 5A and Supporting Information Table S2). Targeted analysis of PC interactome in PLum-AI cells vs. PLum-AD cells revealed dysregulation of proteins involved in cell differentiation, cell proliferation, cell cycle, and apoptosis (Figure 5B and Supporting Information Table S3). In addition, biological process gene ontology (GO) enrichment analysis of the differentially expressed proteins demonstrated enrichment of biological functions in PLum-AI cells that are central to PI3 kinase (Figure 6A) and androgen receptor (Figure 6B) pathways (Supporting Information Tables S4 and S5, respectively).

## 4. Discussion

This work involves the use of LC-MS/MS-based bottom-up proteomics analysis of two murine PC cell lines that represent the sequence of AD-to-AI PC progression, to identify evolutionarily conserved expression changes in PC progression that could serve as potential biomarkers and therapeutic drug targets. We used our previously developed murine PC cell line models (PLum-AD and PLum-AI cells) that signify the development of androgen independence [12] and harbor the same genetic background (*Pten−/−TP53−/−*) [38,39]. Our results identified a total of 683 gene products that were differentially expressed between PLum-AI and PLum-AD cells. Among those, 99 were significantly differentially expressed. Global subnetwork analyses revealed differences in the involvement of protein pathways relevant to PC progression and enrichment of biological functions and pathways in PLum-AI cells that are central to cell migration, cell cycle regulation, cell damage, and cell adhesion among other.

Two major hypotheses have been postulated to decipher the mechanisms underlying progression of PC to CRPC: the adaptive mechanism and the selective mechanism [40]. On one hand, the former suggests that this progression might be highly attributed to gene mutations in PC cells, including AR gene amplifications and mutations, dysregulation of gene expression, and involvement of AR co-regulators such as AR-associated proteins [40]. The androgen receptor (AR) is not only important for normal prostate development, but also promotes PC initiation and growth. Investigating its role in the advancement of PC shows that during the androgen-dependent stage, PC cells rely on AR for growth and survival using several axes [3,4]. Later, and upon the progression of cancer to an androgen-independent stage, affected cells tend to tailor different cellular pathways and mechanisms to survive the androgen-depleted environment. In our study, biological process GO enrichment analysis of the differentially expressed proteins in PLum-AI cells relative to PLum-AD cells revealed enrichment of biological functions that are central to the AR pathway (Figure 6B), supporting the ‘‘adaptation’’ model hypothesis. Indeed, this model proposes that castration-resistant cells originate from genetic mutations of previously androgen-dependent cells during conditions of androgen deprivation [41].

On the other hand, the selective mechanism suggests that pre-existing castration-resistant subclones in primary PC tissues and CSC selection dominate CRPC development [40]. This “clonal selection’’ model suggests that castration resistance emerges from a previously quiescent population of rare castration-resistant cells, such as CSCs that are AR negative, and therefore insensitive to androgen deprivation [41]. This subpopulation of androgen-independent CSCs resides within the tumor bulk and has been associated with PC recurrence [8,10,11,12,42]. Interestingly, our results demonstrated upregulation of a number of proteins in PLum-AI cells that are associated with CSCs, suggesting involvement of these cells with CRPC development. For instance, aldehyde dehydrogenase (ALDH)-2 and phosphoenolpyruvate carboxykinase isoform 2 (PCK2) were upregulated in PLum-AI cells compared to PLum-AD cells. Review of the literature reveals that high expression levels of PCK2 are crucial for the metabolic switch and the maintenance of CSCs in PC [43]. Furthermore, ALDHs have been shown to play an important role in the maintenance and survival of CSCs via promoting chemoresistance [44]. ALDH is commonly known to oxidize acetaldehyde to acetate in the pathway of ethanol metabolism. Several stem cells population were shown to exhibit high ALDH activity, including PC stem cells [44]. High ALDH activity was associated with increased expression of putative PC stem cell markers CD44 and integrin α2β1 and was found to be positively correlated with Gleason score and pathologic stage, and inversely associated with patient survival [45,46]. In this context, biological process GO enrichment analysis of the differentially expressed proteins in PLum-AI cells also demonstrated enrichment of biological functions related to the PI3K pathway, which is critical for PC stem-like cell maintenance as previously illustrated by Dubrovska et al. [47]. Importantly, biological functions central to androgen receptor pathways were also enriched in PLum-AI cells, an example of which is the peptidyl-prolyl cis-trans isomerase (FKBP5), a major player in the androgen signaling pathway in PC [48]. Other proteins that have been found to be significantly highly expressed in PLum-AI relative to PLum-AD cells included Comt. Comt is an enzyme responsible for inactivation of endogenous catecholamines and catechol drugs. Comt enzyme activity has been found to be reduced by 4-fold upon substitution of valine (Val) by methionine (Met) at codon 158, hence contributing to the accumulation of mutagenic catechol compounds leading to PC [49,50].

A critical mechanism that embodies development of metastatic CRPC is epithelial-to-mesenchymal transition (EMT) [17,51,52]. This process marks a key step in the invasion and malignant progression of PC and plays a substantial role in therapeutic resistance to antiandrogens and radiotherapy. During EMT, epithelial cells lose their adhesion molecules and gain a motile mesenchymal phenotype [53]. Particularly, EMT is characterized by loss of E-cadherin and decreased expression of cytokeratins and tight junctions, such as zona occludens and occludin, complemented with an increase in mesenchymal markers such as vimentin and N-cadherin [54], rendering cells capable of invading the extracellular matrix (ECM) and metastasize [55,56]. The role of EMT in PC metastasis has been studied [17,56], revealing significant interplay between EMT-related genes and alterations in signaling pathways involved in prostate organogenesis, such as transforming growth factor-beta (TGF-β) [57], epidermal growth factor receptor (EGFR) [58], IL-6 [59,60], AR variants [61,62], fibroblast growth factor (FGF) [63], and Wnt/β-catenin [64,65]. In our study, biologically statistically significant protein interaction analysis of PLum-AI cells showed enrichment of biological functions and pathways that are central to the EMT process. Indeed, we found upregulation of vimentin (mesenchymal marker) and downregulation of epithelial cell adhesion molecule (EpCAM) (epithelial marker) proteins in PLum-AI cells at the proteomics level and validated them using Western blotting analysis. Fascin, another marker expressed widely in mesenchymal tissues and associated with aggressive PC, was upregulated in PLum-AI cells at the proteomics level [66]. Taken together, these results signify activation of the EMT process in PLum-AI cells that represent advanced CRPC.

## 5. Conclusions

Decoding the molecular networks underlying the progression of the disease from a primary stage to an advanced one is highly warranted to better comprehend the pathobiology of CRPC and unveil its aggressive characteristics. This will ultimately prompt the identification of new potential biomarkers and pave the way for tailored and targeted treatments that are patient-specific [67,68]. It is becoming apparent that this new emerging bioinformatics technology is vital in deciphering mechanistic changes underlying PC and identifying new biomarkers for early diagnosis and predicting patient prognosis of this cancer. Through assessing the proteomic profiles of our previously established PLum-AD and PLum-AI cell lines that represent primary and advanced stages of PC, respectively, and performing a detailed functional proteomics analysis, our results pave the way for understanding the mechanisms exploited in AD-to-AI PC progression. New expression biomarkers and therapeutic targets have been recognized in our study that might help to improve the processes of diagnosing, managing, or even curing the disease. Future studies warrant further validation of these biomarkers at the transcript and protein level.

## Figures and Tables

**Table 1:** Altered differential protein spots in the PLum-AI cells vs. PLum-AD cells. A total of 99 altered differential protein spots were identified in PLum-AI cells as compared to PLum-AD cells under significance less than 0.05. (45 increased and 54 decreased, PLum-AI cells vs. PLum-AD cells).

Protein Accession Number	Identified Protein	Gene Name	Molecular Weight (Daltons)	LogFC	*p*-Value
P29477	Nitric oxide synthase, inducible	Nos2	130,575	Infinity	0.0421
Q4KMM3	Oxidation resistance protein 1	Oxr1	95,912	Infinity	0.0062
Q8BH04	Phosphoenolpyruvate carboxykinase (PEPCK), mitochondrial	Pck2	70,528	Infinity	0.0211
Q60760	Growth factor receptor-bound protein 10	Grb10	70,585	Infinity	0.0005
P24270	Catalase	Cat	59,795	Infinity	0.0370
Q64669	NAD(P)H dehydrogenase (quinone) 1	Nqo1	30,960	Infinity	0.0007
O88587	Catechol O-methyltransferase	Comt	29,486	Infinity	0.0139
P62281	40S ribosomal protein S11	Rps11	18,431	Infinity	0.0244
P21981	Protein-glutamine gamma-glutamyltransferase 2	Tgm2	77,061	4.4671	0.0039
Q9D1A2	Cytosolic non-specific dipeptidase	Cndp2	52,767	3.6410	0.0159
Q9DCX2	ATP synthase subunit d, mitochondrial	Atp5h	18,749	3.1880	0.0049
P28063	Proteasome subunit beta type-8	Psmb8	30,260	3.1819	0.0287
P35282	Ras-related protein Rab-21	Rab21	24,106	3.0182	0.0095
P47738	Aldehyde dehydrogenase, mitochondrial	Aldh2	56,538	2.3290	0.0005
Q9JMH6	Full = Thioredoxin reductase 1	Txnrd1	67,084	2.2227	0.0183
Q99NB9	Splicing factor 3B subunit 1	Sf3b1	145,816	2.1813	0.0045
P47758	Signal recognition particle receptor subunit beta	Srprb	29,579	2.1686	0.0301
Q62351	Transferrin receptor protein 1	Tfrc	85,731	2.0240	0.0258
Q64378	Peptidyl-prolyl cis-trans isomerase FKBP5	Fkbp5	50,966	1.8587	0.0311
Q8VDN2	Full = Sodium/potassium-transporting ATPase subunit alpha-1	Atp1a1	112,982	1.8237	0.0261
Q8R180	ERO1-like protein alpha	Ero1a	54,084	1.7824	0.0028
P26443	Glutamate dehydrogenase 1, mitochondrial	Glud1	61,337	1.7622	0.0313
Q8VDM4	26S proteasome non-ATPase regulatory subunit 2	Psmd2	100,203	1.3712	0.0190
Q9CYA0	Cysteine-rich with EGF-like domain protein 2	Creld2	38,220	1.2856	0.0364
P67778	Prohibitin	Phb	29,820	1.2844	0.0187
Q9R0P3	S-formylglutathione hydrolase	Esd	31,320	1.2832	0.0021
Q9DCT8	Cysteine-rich protein 2	Crip2	22,727	1.2703	0.0017
O88342	WD repeat-containing protein 1	Wdr1	66,407	1.2169	0.0318
P14131	Full = 40S ribosomal protein S16	Rps16	16,445	1.1228	0.0228
Q61553	Fascin	Fscn1	54,508	1.0726	0.0476
P20152	Vimentin	Vim	53,688	1.0105	0.0004
Q64727	Vinculin	Vcl	116,717	0.9370	0.0304
P80317	T-complex protein 1 subunit zeta	Cct6a	58,004	0.9272	0.0335
P17182	Alpha-enolase	Eno1	47,141	0.8210	0.0364
P60335	Poly (rC)-binding protein 1	Pcbp1	37,498	0.8126	0.0037
P70670 (+1)	Nascent polypeptide-associated complex subunit alpha, muscle-specific form	Naca	220,499	0.8122	0.0194
Q922B2	Aspartate–tRNA ligase, cytoplasmic	Dars	57,147	0.8096	0.0454
Q60605	Myosin light polypeptide 6	Myl6	16,930	0.7325	0.0474
P48678	Prelamin-A/C	Lmna	74,238	0.7302	0.0132
Q08288	Full = Cell growth-regulating nucleolar protein	Lyar	43,736	0.7127	0.0244
P05064	Fructose-bisphosphate aldolase A	Aldoa	39,356	0.6559	0.0180
Q9CZ13	Cytochrome b-c1 complex subunit 1, mitochondrial	Uqcrc1	52,852	0.6106	0.0408
P52480	Full = Pyruvate kinase PKM	Pkm	57,845	0.5844	0.0461
Q8VDD5	Myosin-9	Myh9	226,372	0.4277	0.0164
P97384	Annexin A11	Anxa11	54,079	0.3144	0.0315
P68254	14-3-3 protein theta	Ywhaq	27,778	−0.1887	0.0445
P68372	Tubulin beta-4B chain	Tubb4b	49,831	−0.2529	0.0332
Q02053	Ubiquitin-like modifier-activating enzyme 1	Uba1	117,809	−0.4917	0.0040
Q8R1B4	Eukaryotic translation initiation factor 3 subunit C	Eif3c	105,531	−0.6056	0.0123
P50543	Protein S100-A11	S100a11	11,083	−0.6181	0.0341
Q9D819	Inorganic pyrophosphatase	Ppa1	32,667	−0.6647	0.0186
P18760	Cofilin-1	Cfl1	18,560	−0.7070	0.0359
P27546	Microtubule-associated protein 4	Map4	117,429	−0.8789	0.0044
Q8C1B7	Septin-11	Sept11	49,695	−0.8938	0.0365
P30416	Peptidyl-prolyl cis-trans isomerase FKBP4	Fkbp4	51,572	−0.9705	0.0146
Q99MN1	Lysine–tRNA ligase	Kars	67,840	−1.0337	0.0008
P48962	ADP/ATP translocase 1	Slc25a4	32,904	−1.0449	0.0264
P10107	Annexin A1	Anxa1	38,734	−1.0643	0.0202
P13020	Gelsolin	Gsn	85,942	−1.0647	0.0162
Q60932	Voltage-dependent anion-selective channel protein 1	Vdac1	32,351	−1.2973	0.0009
Q60864	Stress-induced-phosphoprotein 1	Stip1	62,582	−1.3133	0.0021
P62827	GTP-binding nuclear protein Ran	Ran	24,423	−1.3401	0.0325
P62830	60S ribosomal protein L23	Rpl23	14,865	−1.3537	0.0023
Q76MZ3	Serine/threonine-protein phosphatase 2A 65 kDa regulatory subunit A alpha isoform	Ppp2r1a	65,323	−1.3762	0.0235
P17879	Heat shock 70 kDa protein 1B	Hspa1b	70,176	-1.3879	0.0209
O55131	Septin-7	Sept7	50,550	−1.4336	0.0409
Q8BGQ7	Alanine–tRNA ligase, cytoplasmic	Aars	106,909	−1.4824	0.0113
Q99K85	Phosphoserine aminotransferase	Psat1	40,473	−1.4994	0.0014
Q6IRU2	Tropomyosin alpha-4 chain	Tpm4	28,468	−1.5363	0.0019
P62814	V-type proton ATPase subunit B, brain isoform	Atp6v1b2	56,551	−1.5772	0.0284
P99029	Peroxiredoxin-5, mitochondrial	Prdx5	21,897	−1.7034	0.0397
Q9Z110	Delta-1-pyrroline-5-carboxylate synthase	Aldh18a1	87,266	−1.7114	0.0488
O70400	PDZ and LIM domain protein 1	Pdlim1	35,774	−1.7682	0.0055
P08003	Protein disulfide-isomerase A4	Pdia4	71,982	−1.7998	0.0218
Q78PY7	Staphylococcal nuclease domain-containing protein 1	Snd1	102,088	−1.9226	0.0089
Q3THE2 (+1)	Myosin regulatory light chain 12B	Myl12b	19,779	−1.9663	0.0019
Q9DAW9	Calponin-3	Cnn3	36,429	−1.9714	0.0138
P47226	Testin	Tes	47,983	−2.1001	0.0061
P19096	Fatty acid synthase	Fasn	272,428	−2.1356	0.0143
Q8VH51	RNA-binding protein 39	Rbm39	59,407	−2.1700	0.0432
P16546	Spectrin alpha chain, non-erythrocytic 1	Sptan1	284,597	−2.1906	0.0004
O55023	Inositol monophosphatase 1	Impa1	30,436	−2.2878	0.0041
P34022	Ran-specific GTPase-activating protein	Ranbp1	23,596	−2.3426	0.0192
Q9CZY3	Ubiquitin-conjugating enzyme E2 variant 1	Ube2v1	16,355	−2.5164	0.0385
Q9JKF1	Ras GTPase-activating-like protein IQGAP1	Iqgap1	188,742	−2.5447	0.0001
Q9EQH3	Vacuolar protein sorting-associated protein 35	Vps35	91,713	−2.6401	0.0140
Q69ZN7	Myoferlin	Myof	233,324	−2.7480	0.0041
Q00PI9	Heterogeneous nuclear ribonucleoprotein U-like protein 2	Hnrnpul2	84,940	−infinity	0.0466
Q6P1B1	Xaa-Pro aminopeptidase 1	Xpnpep1	69,591	−infinity	0.0029
Q8JZK9	Hydroxymethylglutaryl-CoA synthase, cytoplasmic	Hmgcs1	57,569	−infinity	0.0493
Q8VI94	2′-5′-oligoadenylate synthase-like protein 1	Oasl1	59,088	−infinity	0.0024
Q64282	Interferon-induced protein with tetratricopeptide repeats 1	Ifit1	53,737	−infinity	0.0054
Q8CAY6	Acetyl-CoA acetyltransferase, cytosolic	Acat2	41,298	−infinity	0.0406
Q920E5	Farnesyl pyrophosphate synthase	Fdps	40,582	−infinity	0.0080
Q99JW5	Epithelial cell adhesion molecule	Epcam	35,019	−infinity	0.0027
P70677	Caspase-3	Casp3	31,475	−infinity	0.0054
Q62393	Tumor protein D52	Tpd52	24,313	−infinity	0.0036
P61967	AP-1 complex subunit sigma-1A	Ap1s1	18,733	−infinity	0.0498
Q9CR51	V-type proton ATPase subunit G 1	Atp6v1g1	13,724	−infinity	0.0430

## Supplementary Materials

The following are available online at https://www.mdpi.com/2075-4426/10/3/83/s1, Figure S1: Densitometry readings/intensity ratio of each band in addition to whole blot (uncropped blots) in PLum-AI vs. PLum-AD cell samples; Table S1: Altered differential protein spots in the PLum-AI cells vs. PLum-AD cells. A total of 99 altered differential protein spots were identified in PLum-AI cells as compared to PLum-AD cells under significance less than 0.05. (45 increased and 54 decreased, PLum-AI cells vs. PLum-AD cells); Table S2: Biologically statistically significant protein interaction analysis in PLum-AI cells vs. PLum-AD cells; Table S3: Targeted analysis of prostate cancer interactome in PLum-AI cells vs. PLum-AD cells, Table S4: Biological process gene ontology (GO) enrichment analysis of the differentially expressed proteins in PLum-AI cells vs. PLum-AD cells demonstrated enrichment of biological functions in PLum-AI cells that are central to PI3 kinase pathway; Table S5: Biological process gene ontology (GO) enrichment analysis of the differentially expressed proteins in PLum-AI cells vs. PLum-AD cells demonstrated enrichment of biological functions in PLum-AI cells that are central to the androgen receptor (AR) pathway.
