# Supplementary figures and images for: Protein Expression Analysis of an In Vitro Murine Model of Prostate Cancer Progression: Towards Identification of High-Potential Therapeutic Targets
Source: J Pers Med. 2020 Aug 10;10(3):83. doi: 10.3390/jpm10030083 (PMC7565308; doi:10.3390/jpm10030083)

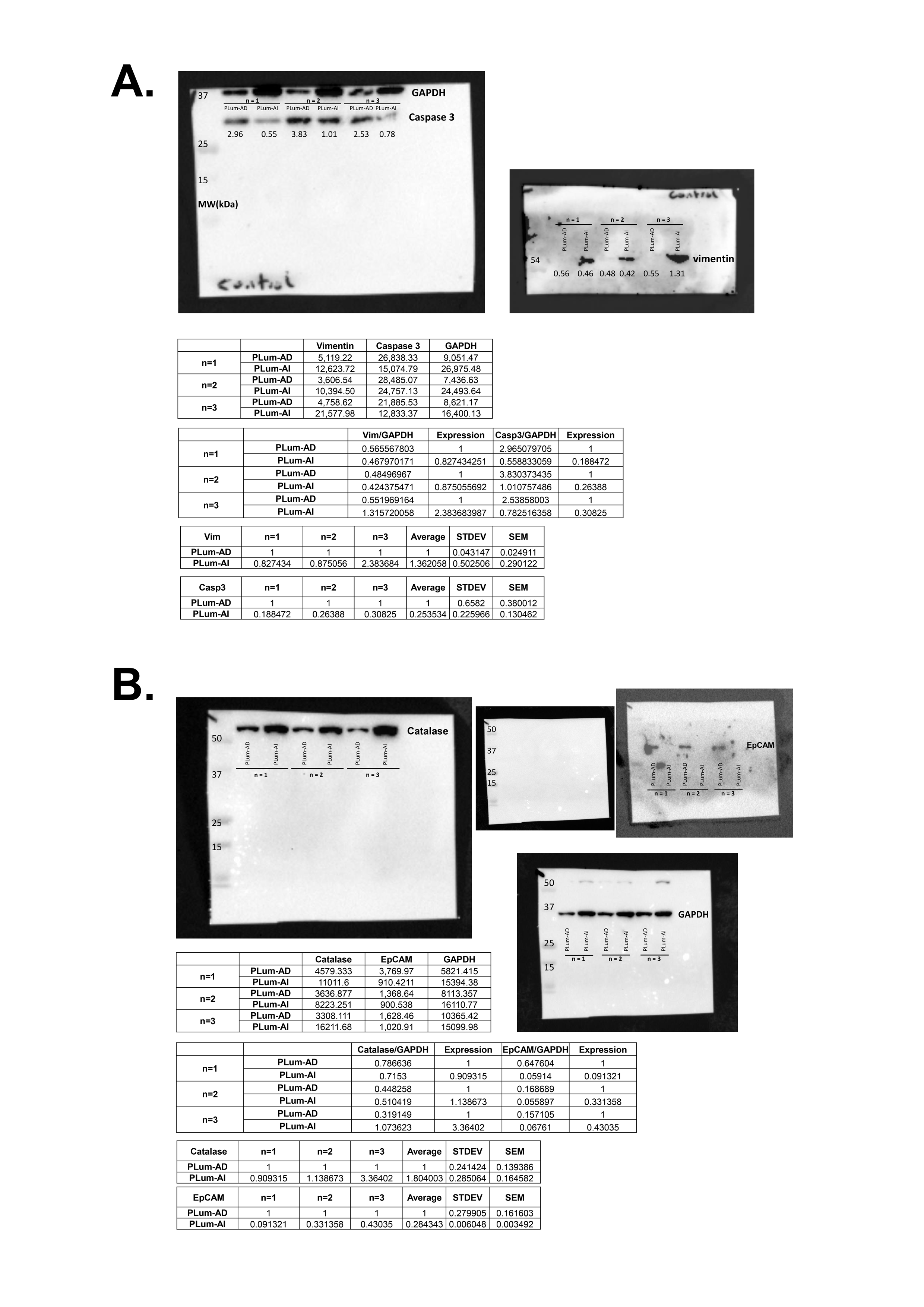

Supplement: Supplementary file 1 [file jpm-10-00083-s001.zip › Supplementary Figure 1.tif]
